# A clinical pathway for the management of difficult venous access

**DOI:** 10.1186/s12912-017-0261-z

**Published:** 2017-11-17

**Authors:** Vanno Sou, Craig McManus, Nicholas Mifflin, Steven A. Frost, Julie Ale, Evan Alexandrou

**Affiliations:** 1 Liverpool Hospital, Liverpool, Australia; 2 Western Sydney University, Building EB, Ground Level Room 44, Parramatta South Campus, Locked Bag 1797, Penrith South. DC 1797, NSW 2751 Australia; 3 Alliance for Vascular Access Teaching and Research Group, Menzies Health Institute Queensland, Griffith University, Nathan, Australia; 4 Simpson Centre for Health Services Research and Centre for Applied Nursing Research, Sydney, Australia; 5 South Western Sydney Clinical School & Ingham Institute of Applied Medical Research, University of New South Wales, Sydney, Australia

**Keywords:** Intravenous catheter, Peripheral venous catheter, Ultrasound

## Abstract

**Background:**

Many patients are admitted to hospital with non-visible or palpable veins, often resulting in multiple painful attempts at cannulation, anxiety and catheter failure. We developed a difficult intravenous pathway at our institution to reduce the burden of difficult access for patients by increasing first attempt success with ultrasound guidance. The emphasis was to provide a solution for hospitalised patients after business hours by training the after-hours clinical support team in ultrasound guided cannulation.

**Methods:**

Inception cohort study of patients referred to the after-hours clinical support team including outcomes such as number of attempts at cannulation before and after referral, insertion site, type of device inserted and recorded pain score for attempts prior to referral and for attempts by the after-hours clinical support team.

**Results:**

Between January and December 2016, 379 patients were referred to the after-hours clinical support team for placement of a peripheral intravenous catheter under ultrasound guidance. The median number of unsuccessful attempts before referral was 2 (IQR 2, 4), this ranged between 1 attempt to 10 attempts compared to only 1 attempt (IQR 1, 1, *p* < 0.001) with no more than 2 attempts in total by the after-hours clinical support team. The first time success rate by the after-hours clinical support team was 93% (*n* = 348). The median pain score for attempts with ultrasound use was 2/10 (IQR 1–3) compared to 7/10 (IQR 5–9) for previous attempts without ultrasound (*p* < 0.001).

**Conclusion:**

The use of ultrasound guidance for peripheral intravenous catheter insertion by the after-hours clinical support team for patients with difficult venous access has been successful at our institution with 9 out of every 10 catheters inserted at first attempt with significantly lower recorded pain scores.

## Background

It is estimated that over half of all patients admitted to hospital require the insertion of a peripheral intravenous catheter (PIVC) for the administration of fluids and parenteral medications [1]. It is the most common invasive clinical procedure performed in hospitals worldwide [2, 3]. Over a third of adults and up to half of the children that present to hospital who require a PIVC, are reported to have difficult venous access (DiVA) [4, 5].

Difficult venous access is characterised by non-visible and non-palpable veins where a highly experienced operator is required with the use of technological aids to insert a vascular device [6]. Patients with chronic and complex disease, who have a history of intravenous drug use, are obese or malnourished or who have received chemotherapy, are cohorts known to suffer from DiVA [5–7].

Patients with DiVA may undergo multiple, painful attempts to gain peripheral venous access [7]. Importantly, there can be many clinical implications from DiVA, namely: a delay in diagnosis, where important laboratory tests are required; delay in the commencement of treatment, or missed medication doses; and, if severe, can require escalation for insertion of a central venous access device (CVAD) [5, 8, 9].

It has been well described that multiple attempts at cannulation, and the placement of PIVCs in high flexion areas such as the elbow or wrist (which is typical among DiVA patients) increases the risk of phlebitis, thrombosis, and catheter related infection - all of which lead to premature device failure [10, 11]. This in turn, results in further painful, and often unsuccessful attempts to gain alternate peripheral venous access, leading to vessel damage and venous depletion [12].

Ultrasound guidance for the insertion of CVADs has become standard practice, and has been demonstrated in a number of investigations to reduce insertion attempts, and mitigate serious procedural complications such as pneumothorax and accidental arterial puncture [13–15]. There is an emerging trend for the use of this technology to aid in the insertion of PIVCs for patients with DiVA [16, 17]. A recent systematic review, of six randomised control trials, testing the effectiveness of ultrasound guidance versus traditional approaches to cannulation found a near fourfold increase in success rates when ultrasound was used for peripheral cannulation by experienced operators [8].

In our institution, as with other modern hospitals that treat large numbers of patients with acute and chronic diseases, an increasing number of patients were presenting with DiVA. Medical teams, nursing staff and the after-hours clinical support team (AHCST – consisting of clinical nurse consultants and clinical nurse specialists), had difficulty in placing PIVCs using traditional methods (particularly in the evening hours). Consequently, patients would be referred to the hospital’s central venous access service for the placement of a vascular device. Although highly skilled, the central venous access service did not have the capacity to facilitate the entire hospital in a timely fashion. As a result, patients would undergo a number of painful and sometimes unsuccessful attempts at cannulation until definitive access was obtained.

In an attempt to reduce the burden of DiVA for patients and treating teams, the AHCST along with the central venous access service, developed a DiVA pathway that provided better direction for clinical teams to manage patients with difficult vasculature. The DiVA pathway aims to reduce delays in intravenous therapy, reduce the number of insertion attempts and provide a guide as to the best vascular device tailored to the patient’s care. Importantly, the DiVA pathway, is a strategy to maintain vessel health and preservation for all patients but particularly for those with chronic and complex disease [12]. The aim of this study was to describe and evaluate the success of the AHCST utilising ultrasound guidance to place peripheral catheters for patients suffering DiVA.

## Methods

### Study design and setting

The setting for this Inception cohort study was an 877-bed tertiary referral university hospital, in the South West of Sydney, Australia. The emergency department has approximately 80,000 presentations annually, with a similar number of hospital overnight stays. The DiVA pathway involved a hospital wide approach, which included a briefing to the hospital executive on the problem facing clinical teams, and the DiVA pathway solution. The primary aspect of the project was training the AHCST in ultrasound guidance for PIVC insertion until a more definitive vascular access could be attained by the central venous access service.

Training for the AHCST involved didactic sessions with the central venous access service, completion of a learning package that included a short examination, supervised simulated ultrasound cannulation practice with medium fidelity models, and finally successful supervised ultrasound guided PIVC insertions. Successful credentialing with ultrasound was based on both the trainers and trainees feeling comfortable with the clinical skill; on average this took 15 successful cannulation attempts with ultrasound.

**Fig. 1:**
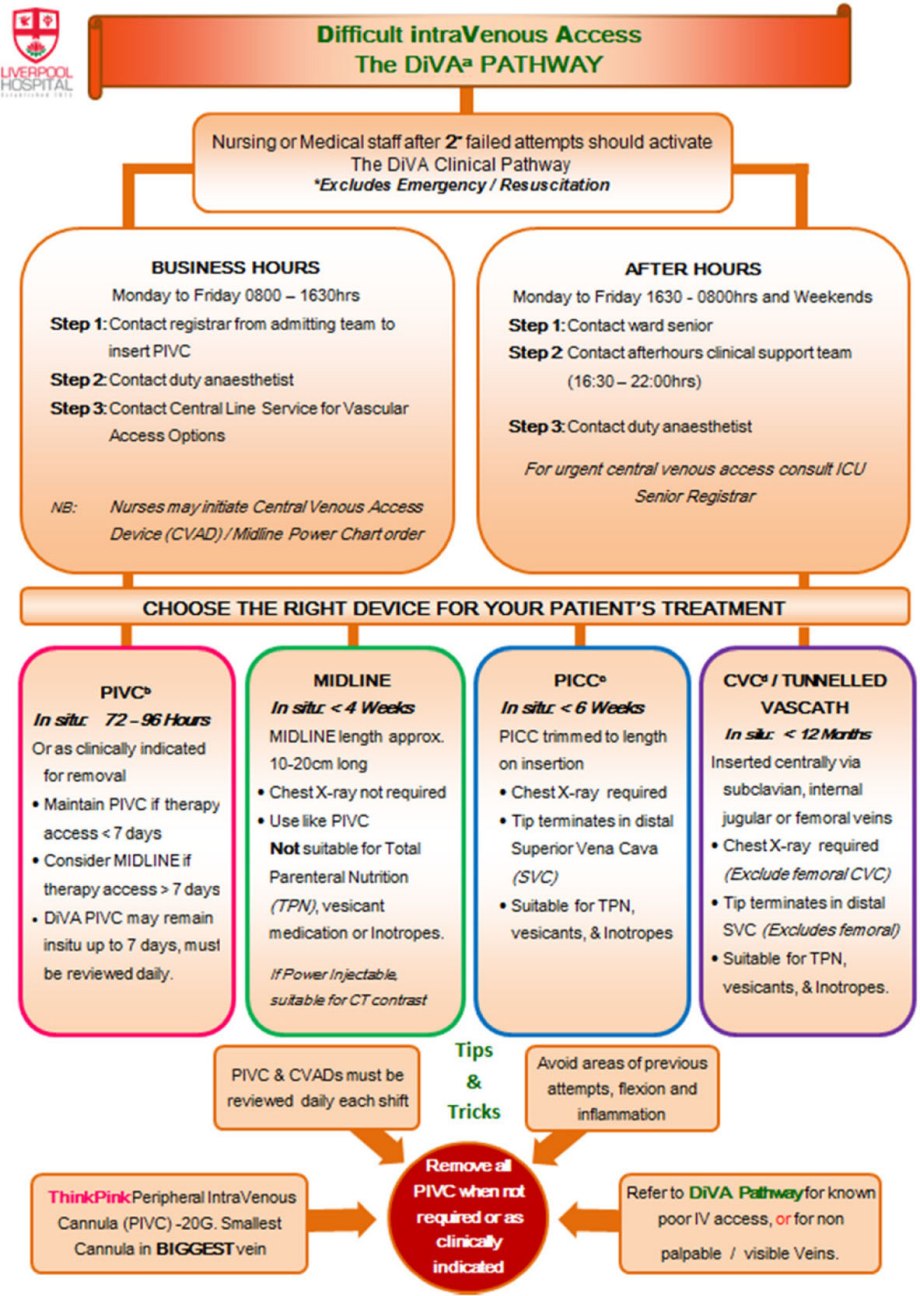
Difficult intravenous access pathway. The flow diagram illustrates the process of contacting trained personnel during business hours and after hours for treating teams and includes a synopsis of vascular devices available. **a** *DiVA* Difficult venous access, **b** *PIVC* Peripheral intravenous catheter, **c** *PICC* Peripherally inserted central catheter, **d** *CVC* Central venous catheter

**Fig. 2:**
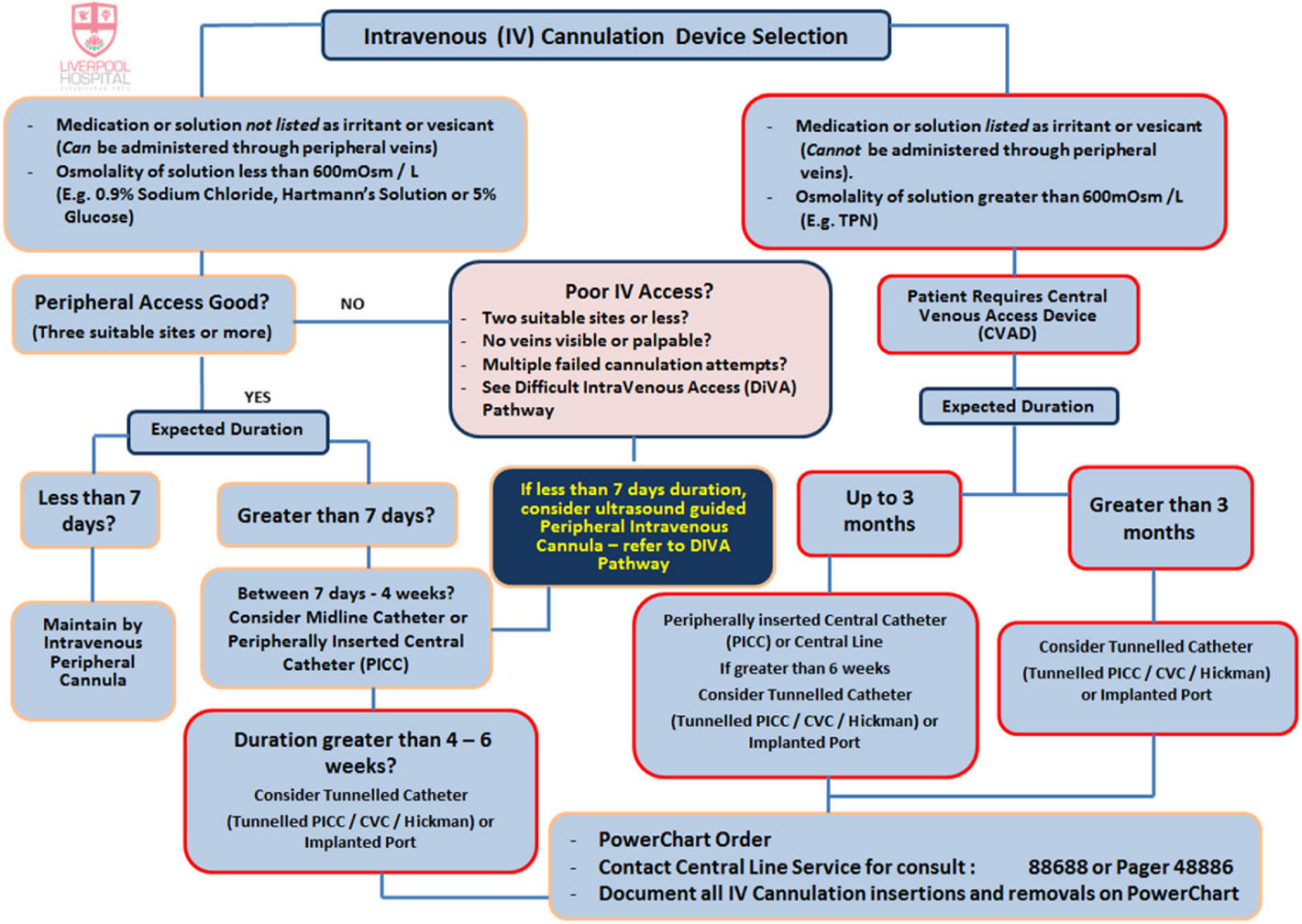
IV Access Vascular Device Decision Tree. The clinical pathway illustrates best choice of vascular access device tailored to patient treatment when considering intravenous fluid, intravenous medication, vascular assessment and length of anticipated dwell

The project also involved the development of two pathways: (1) the DiVA flowchart that directed clinical staff who to contact during business hours and afterhours (Fig. 1); and, (2) A device selection algorithm based on clinical evidence and local institution consensus (Fig. 2) [18, 19].

Patients admitted to our hospital should, by policy, have no more than two attempts at peripheral cannulation by any one individual, after which senior staff members are notified. If senior staff attempt and fail, then the DiVA pathway is activated. The DiVA pathway can also be activated without prior attempts for patients with known difficult access or where staff has deemed the patient to be difficult. All PIVCs inserted by the AHCST were placed using an aseptic technique and not routinely replaced but removed when clinically indicated or when definitive access was attained.

We reviewed data that was entered into a purpose built Microsoft Access database (Microsoft Office Professional Plus 2010, Version 14.0.7128.5000) contemporaneously for this project. Variables included patient demographics, reason for PIVC insertion, size and type of device inserted, number of attempts before referral, and with ultrasound guidance. The primary outcomes of interest for this study were: 1) the number of cannulation attempts required with ultrasound guidance compared to the number of attempts prior to referral and, 2) the reported pain score (using a numeric rating scale: 0–10) [18] with ultrasound guided peripheral cannulation compared to attempts without ultrasound. Patient medical records were also reviewed where necessary.

Local Human Research Ethics Committee approval was granted (reference number: LNR/15LPOOL/518) prior to the commencement of the study. The Standards for Quality Improvement Reporting Excellence version 2.0 (SQUIRE 2.0) guidelines for reporting improvements in healthcare were followed and results are presented following these recommendations [19].

### Statistical analysis

Descriptive statistics are presented as mean (standard deviation, SD), or median (Inter Quartile Range, IQR) numbers and proportions where appropriate. Various characteristics where compared using a two sample t-test, this included comparing difference between the number of attempts at cannulation pre and post ultrasound. Non parametric tests were also used including the Wilcoxon signed-rank test to assess whether pain scores differed with cannulation attempts before referral to the AHCST and attempts by the AHCST. The cut-off for statistical significance was calculated at *p* < 0.05. All analyses were undertaken using the R language for statistical analysis (R Core Team Vienna, Italy).

## Results

The characteristics of males and females referred to the AHCST are presented in Table 1. Between January and December 2016, a total of 379 patients were referred for placement of a PIVC under ultrasound guidance. The average age of patients was 66 years (SD 17), and of the 379 patients referred to DiVA 165 (43.5%) were males. Although height and weight were statistically significant between genders (*p* < 0.001), the BMI was not and ranged from 14.7 to 80.6 (kg/m^2^) with a median of 28 (IQR 23, 34).

**Table 1:** Characteristics of patients referred to after-hours clinical support team

	Males (*n* = 165)	Females (*n* = 214)	Combined (*n* = 379)	*p*-value
Age (y), mean (SD)^a^	67 (16)	66 (18)	66 (17)	0.92
Height (cm), mean (SD)	170 (8.5)	159 (8.0)	164 (9.6)	< 0.001
Weight (kg), median (IQR)	80 (68,100)	72 (56, 85)	75 (60, 90)	< 0.001
BMI^b^ (kg/m^2^), median (IQR) [BMI range, max - min]	28 (24, 34) [14.7–80.6]	28 (22, 33) [15.1–70.3]	28 (23, 34) [14.7–80.6]	0.620
Specialty, n (%)				0.730
Medical	71 (43)	82 (38)	153 (40)	
Surgical	38 (23)	58 (27)	96 (25)	
Haematology/Oncology	23 (14)	27 (13)	50 (13)	
ICU/CCU^c^	24 (14)	23 (11)	47 (12	
Aged care	6 (4)	17 (8)	23 (6)	
Maternity	0	3(1)	3 (1)	
Paediatrics	1 (1)	1	2 (1)	
Other	2 (1)	3 (1)	5 (1)	
Previous attempts, median (IQR)^d^ [Range, max – min]^e^	3 (2, 4) [1, 10]	2 (2,4) [1, 10]	2 (2,4) [1, 10]	0.780
Pain score (0–10) during last attempt, median (IQR) [Range, max - min]	6 (4,8) [2, 10]	7 (6, 10) [3, 10]	7 (5, 9) [2, 10]	0.130

^a^ *SD* Standard deviation, ^b^ *BMI* Body mass index, ^c^ *ICU/CCU* Intensive care unit/coronary care unit, ^d^ *IQR* Inter quartile range, ^e^ *Max – min* Maximum – minimum

The median number of attempts by clinicians before referral to the AHCST was 2 (IQR 2, 4), this ranged from a single attempt and up to 10 attempts for both genders (Table 1). Reported pain scores for catheter insertion attempts prior to referral were numerically high, with a combined median pain score of 7 (IQR 5, 9). Referrals for PIVC insertion were predominantly for general medical (*n* = 153, 40%) and general surgical (*n* = 96, 25%) patients, and collectively comprised 3 in every 5 PIVC insertion referrals.

The characteristics of the AHCST interventions are presented in Table 2. The basilic veins in the upper arms were the preferred site of cannulation for both males and females, they comprised nearly 70% of all cannulations (*n* = 262) with over half the devices being 20G PIVCs (*n* = 219, 58%). Devices ranged from simple safety cannulas, integrated devices with extension sets and accelerated seldinger devices. The minimum length used was 45 mm and the most common reason for a PIVC was for intravenous medication and fluids (*n* = 284, 75%).

**Table 2:** Characteristics of after-hours clinical support team interventions

	Males (*n* = 165)	Females (*n* = 214)	Combined (*n* = 379)	*p*-value
Indication for PIVC^a^, n (%)				
Medications/Fluids	118 (72)	166 (78)	284 (75)	0.240
CT^b^ contrast	8 (5)	12 (6)	20 (5)	
Medical Emergency	2 (1)	2 (1)	4 (1)	
Blood transfusion	7 (4)	2 (1)	9 (2)	
Other	30 (18)	32 (15)	62 (16)	
Insertion site, n (%)				0.560
Basilic	109 (66)	153 (71)	262 (69)	
Cephalic	16 (10)	18 (8)	34 (9)	
Medial cubital	22 (13)	22 (10)	44 (12)	
Antecubital	14 (8)	16 (7)	30 (8)	
Device gage, n (%)				0.400
20 g PIVC	92 (56)	127 (59)	219 (58)	
20 g midline	1 (1)	0 (0)	1 (1)	
18 g PIVC	56 (34)	66 (31)	122 (32)	
18 g midline	0 (0)	1 (1)	1 (1)	
22 g PIVC	11 (7)	8 (4)	19 (5)	
24 g PIVC	0 (0)	2 (1)	2 (1)	
Other	5 (3)	7 (3)	12 (4)	
First attempts success (%)	153 (95)	195 (92)	348 (93)	0.240
No. of attempts, median (IQR)^c^ [Range, min – max]^d^	1 (1,1) [1, 2]	1 (1,1) [1, 2]	1 (1,1) [1, 2]	0.100
Pain score, median (IQR)^c^ [Range, min - max]	2 (1, 3) [1–7]	1 (1,3) [0–8]	2 (1,3) [0–8]	0.600
Procedural time in minutes, mean (SD)^e^	13.6 (5.3)	13.7 (6.4)	13.6 (6.0)	0.850

^a^ *PIVC* Peripheral intravenous catheter, ^b^ *CT* Computerised tomography, ^c^ IQR Inter quartile range ^d^ *Max – min* Maximum – minimum, ^e^ *SD* Standard Deviation

First time success for cannulation with ultrasound by the AHCST for both genders was 93% (*n* = 348). The median number of attempts was 1 (IQR 1, 1) with no more than 2 attempts required to gain venous access. The average time for an ultrasound guided cannulation for both men and women (time measured from needle penetrating skin to application of occlusive dressing) by the AHCST was 13.6 min (SD 6.0) (Table 2).

We found a significant difference in pain scores prior to AHCST referral (Table 3). The median pain score for cannulation attempts prior to referral was threefold higher (7/10, IQR 5, 9) compared to cannulation undertaken by the AHCST (2/10, IQR 1, 3, *p* < 0.001). A significant difference was also found between number of cannulation attempts before (2, IQR 2, 4) and after (1, IQR 1, 1) AHCST referral (*p* < 0.001).

**Table 3:** Comparison of cannulation attempts and pain score prior

	Prior to referral	AHCST^a^ referral	*p*-value
No. of attempts, median (IQR)^b^	2 (2, 4)	1 (1, 1)	< 0.001
Pain score, median (IQR)	7 (5, 9)	2 (1, 3)	< 0.001

^a^ AHCST – after-hours clinical support team, ^b^ *IQR* Inter quartile range

## Discussion

The insertion of PIVCs is essential for many therapies. There are a number of patients that present to hospital who have difficult venous access where multiple attempts may be required, often by multiple personnel within the health care facility. This can have deleterious effects on patient care that include missed medication doses, significant pain and increased anxiety as well as increased risk of premature device failure [17, 20, 21].

Previous studies have shown variability in success with use of ultrasound, however good training, along with procedural experience with ultrasound has been shown to be an influencing factor for first time cannulation success [20, 22]. The results of our DiVA pathway emulate previously published studies where first time cannulation success was higher (greater than 80%) when ultrasound was used by trained, experienced operators for this cohort [16, 20, 23]. The use of ultrasound technology can reduce procedural complications, procedural time as well as reducing device failure [8, 24].

The basilic vein in the upper arm was the preferred vessel for cannulation by the AHCST and accounted for nearly 70% of all PIVCs placed. The basilic vein is the preferred vessel for midline and peripherally inserted central catheters (PICCs) as the internal diameter is typically big enough to accommodate a large bore device and is not an area of high flexion [25, 26]. The choice of this vessel may have been due to vessels in the forearm assessed to be inadequate for cannulation, thrombosed or depleted. It could also be due to the use of longer length devices permitting cannulation of this larger vessel for intravenous therapy.

The average time taken for ultrasound guided cannulation by the AHCST was 13.6 min (SD 6.0 min). This was efficient compared to published literature for both traditional and ultrasound approaches for peripheral cannulation [22, 27]. The training and experience of the AHCST was likely a contributing factor, ultrasound guided PIVC insertion, in trained hands has been shown to be twice as fast than the traditional approach for patients with DiVA, improving organisational efficiency and time to treatment [20].

Patients reported a much higher level of pain with cannulation attempts prior to referral for ultrasound guidance, some experienced severe pain with nearly a quarter (23.3%) of patients reporting a pain score of 10/10. This significant finding illustrates the impact of needle pain which can cause anxiety, phobia and significantly reduce patient satisfaction with the health care facility [21]. In a systematic review and network meta-analysis of pain relief for peripheral cannulation in adults, pain was successfully controlled with the application of local anaesthesia in a number of studies [28]. The use of topical or subcutaneous anaesthesia should be considered when placing devices in patients who suffer DiVA [29, 30].

This study needs to be considered within the context of a number of limitations. First, our study is an inception cohort of patients that by design can be subject to numerous biases and confounding. Not all patients suffering from DiVA were referred to the AHCST and other avenues were used (including the hospital central venous access service and anaesthetic department) for placement of a PIVC. As such this sample is not a reflection of all DiVA patients in our institution. Recall bias of pain from cannulation attempts prior to ultrasound insertion may have influenced pain score results. Measurement error may have also influenced average cannulation time (but this would be minor given the similarity in measurement results between genders). Data collection for this project was undertaken contemporaneously which included data entry into a purpose built database.

Our study adds to the growing literature that DiVA is becoming a burden to patients and clinicians worldwide and further work is required on this emerging phenomenon. In our local health district, we have developed clinical alerts on the electronic medical record informing clinicians of patients suffering from DiVA. We have found early identification of patients with DiVA requires an organisational approach so that assessment and management is undertaken as soon as possible to reduce multiple, painful attempts to gain venous access.

## Conclusion

The use of ultrasound guidance for PIVC insertion by the AHCST for patients suffering from DiVA has been successful at our institution. Nearly all patients referred to the AHCST for PIVC insertion with ultrasound guidance had their device inserted first attempt in a timely manner. Pain scores were also significantly lower with ultrasound guidance compared to attempts without ultrasound.

## Abbreviations

AHCST: After hours clinical support team
CVAD: Central venous access device
CVAS: Central venous access service
DiVA: Difficult venous access
PIVC: Peripherally intravenous catheter
